# Supplementary material for: Dynamic Analysis of Physicochemical Properties and Polysaccharide Composition during the Pile-Fermentation of Post-Fermented Tea
Source: Foods. 2022 Oct 26;11(21):3376. doi: 10.3390/foods11213376 (PMC9657414; doi:10.3390/foods11213376)
Supplement: Supplementary file 1 [file foods-11-03376-s001.zip › foods-1956927-supplementary.pdf]

Supplementary Table S1 Content of physical and chemical components during the pile-fermentation of post-fermented tea (*n*=3, %).

Samples	W-0	W-1	W-2	W-3	W-4	W-5	W-6	W-7	C-1
Tea polyphenols	10.83±0.34^{a}	10.37±0.65^{a}	8.44±0.50^{b}	7.62±0.44^{c}	6.59±0.72^{d}	4.44±0.24^{f}	4.78±0.37^{f}	5.21±0.20^{e}	4.63±0.31^{f}
Catechin	7.24±0.08^{a}	6.27±0.02^{b}	5.94±0.09^{c}	5.03±0.12^{c}	3.08±0.02^{d}	3.00±0.36^{d}	3.12±0.45^{d}	3.40±0.26^{d}	3.20±0.62^{d}
Flavone	3.10±0.11^{a}	2.86±0.09^{a}	1.90±0.08^{c}	2.47±0.10^{b}	1.42±0.06^{c}	1.75±0.07^{c}	1.68±0.07^{c}	2.13±0.09^{b}	2.02±0.08^{c}
Theabrownine	4.23±0.33^{d}	5.29±0.33^{c}	5.75±0.29^{c}	5.87±0.33^{c}	6.81±0.57^{b}	6.92±0.60^{b}	6.57±0.33^{b}	6.69±0.60^{b}	9.62±0.29^{a}
Amino acid	1.59±0.02^{a}	1.34±0.04^{b}	1.26±0.06^{b}	1.52±0.01^{a}	1.22±0.07^{b}	1.15±0.21^{b}	1.18±0.13b	1.07±0.04^{b}	0.87±0.04^{c}
Caffeine	1.30±0.04^{b}	1.33±0.00^{b}	1.34±0.00^{b}	1.45±0.03^{b}	1.42±0.02^{b}	1.58±0.01^{b}	1.82±0.00^{a}	1.38±0.00^{b}	1.56±0.03^{b}
Soluble sugar	3.73±0.04^{d}	4.46±0.07^{c}	5.02±0.05^{b}	3.62±0.04^{d}	7.02±0.07^{a}	4.05±0.04^{c}	5.05±0.03^{b}	4.85±0.04^{c}	4.65±0.03^{c}
Tea polysaccharide	3.13±0.14^{g}	5.62±0.08^{f}	12.17±0.06^{e}	16.53±0.08^{c}	17.11±0.09^{b}	18.26±0.07^{a}	16.51±0.10^{c}	15.56±0.06^{d}	15.42±0.03^{d}
Total protein	9.98±0.09^{a}	9.76±0.02^{a}	9.38±0.10^{a}	9.39±0.04^{b}	9.01±0.08^{b}	8.86±0.03^{b}	8.84±0.04^{b}	8.97±0.06^{b}	9.04±0.11^{b}
